# A Redundant System of Thioredoxin and Glutathione Is Essential for Pancreatic Acinar Integrity

**DOI:** 10.1016/j.jcmgh.2025.101627

**Published:** 2025-09-02

**Authors:** Henrik Einwächter, Bailing Li, Michaela Aichler, Mariana Rickmann, Nirav Florian Chhabra, Rupert Oellinger, Markus Brielmeier, Roland M. Schmid

**Affiliations:** 1 Department of Medicine II, Klinikum Rechts der Isar, School of Medicine, Technical University of Munich, Munich, Germany; 2 Core Facility Laboratory Animal Services, Helmholtz Zentrum München - German Research Center for Environmental Health GmbH, Neuherberg, Germany; 3 Institute of Molecular Oncology and Functional Genomics, School of Medicine, Technical University of Munich, Munich, Germany

**Keywords:** Acute Pancreatitis, Glutathione, Thioredoxin

## Abstract

**Background & Aims:**

Oxidative stress and antioxidant defense mechanisms have long been implicated in the pathogenesis of acute pancreatitis (AP). However, there is a notable lack of in vivo experimental evidence clarifying their precise role.

**Methods:**

We generated and analyzed mice with a pancreas-specific deletion of *Txnrd1* (*Txnrd1*^Δpanc^). AP was induced in these mice using cerulein injections. Pancreatic tissue was subsequently analyzed using immunoblotting, histology, immunohistochemistry, RNA sequencing, and biochemical assays.

**Results:**

*Txnrd1*^Δpanc^ mice exhibited normal growth, pancreatic weight, histology, and pancreatic function comparable to controls, although they experienced a slightly more severe course of AP. An increase in glutathione levels and upregulation of components within the glutathione system were observed in these mice. However, depletion of the glutathione pool led to pancreatic necrosis, followed by regeneration. When glutathione depletion was combined with AP, *Txnrd1*^Δpanc^ mice suffered a profound and permanent loss of acinar tissue.

**Conclusions:**

These findings indicate that the response to AP is closely linked to alterations in antioxidant systems. The thioredoxin and the glutathione systems appear to perform overlapping protective roles in safeguarding acinar cells during AP. A simultaneous disruption of both systems proves detrimental to pancreatic integrity during acute pancreatitis.

**Summary**

During acute pancreatitis, antioxidant systems significantly change, and *Txnrd1* is highly upregulated. *Txnrd1* deletion results in compensatory upregulation of glutathione. Glutathione depletion alone does not cause a worse acute pancreatitis, but inhibition of both systems is detrimental for the pancreas.

This article has an accompanying editorial.

Acute pancreatitis (AP) is a condition characterized by the activation of intracellular proteases. In up to 20% of cases, AP leads to extensive organ necrosis, triggering a systemic inflammatory response that can progress to multiple organ failure. The mortality rate for patients with this severe form of the disease is approximately 20%. Although risk factors such as alcohol abuse, gallstones, and hypertriglyceridemia have been identified, the exact mechanisms driving inflammation and determining clinical severity remain poorly understood.

Oxidative stress, defined as an imbalance between production of reactive oxygen species (ROS) and the body’s antioxidant defenses, has been implicated in the pathogenesis of AP since 1984.^1^ It was first proposed that increased capillary permeability, an early feature of AP, might result from ROS activity. Since then, the role of oxidative stress in AP has been the subject of considerable debate (for review, see ^2^). Both experimental and clinical studies have produced conflicting results.

There are several antioxidant systems in the cell. Enzymatic antioxidants can be divided into 2 major categories: metalloenzymes (eg, catalase and superoxide dismutase) and the thiol-dependent systems comprising the thioredoxin and glutathione (GSH) pathways.

Thioredoxins are small proteins that reduce disulfides in oxidized peroxiredoxins, methionine sulfoxide reductases, and ribonucleotide reductases. They can also reduce oxidized transcription factors. In turn, oxidized thioredoxin is regenerated by thioredoxin reductases, which rely on nicotinamide adenine dinucleotide phosphate (NADPH) as an electron donor.

The most abundant cellular antioxidant thiol is GSH, synthesized in a 2-step process catalyzed by glutamate cysteine ligase and by GSH synthetase. GSH serves as an electron donor in reactions catalyzed by GSH peroxidases, during which it is oxidized to glutathione disulfide (GSSG). GSSG is subsequently reduced back to GSH by GSSG reductase, a process that also requires NADPH.

In experimental models of pancreatitis, glutathione depletion within the first 4 to 8 hours has been identified as a critical factor in the disease’s pathogenesis of pancreatitis.^3^ This underscores the importance of antioxidant defenses in mitigating the early stages of AP.

## Results

### Antioxidant Systems Undergo Significant Changes During AP

**Figure 1:**
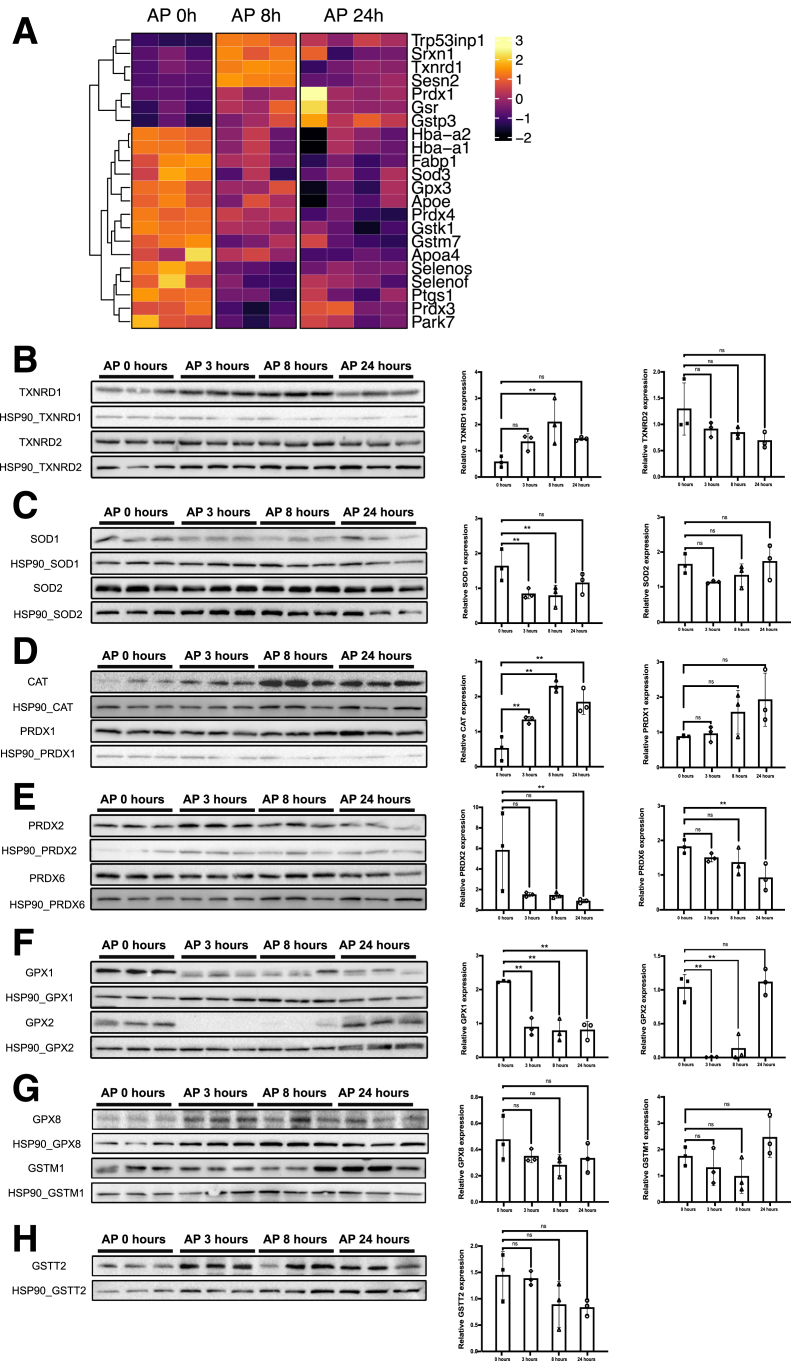
**Protein expression of components of main antioxidant systems during pancreatitis.** (*A*) Heat map for expression levels of significantly regulated antioxidant genes during AP. (*B*) Immunoblot for TXNRD1 and TXNRD2 during AP (*left*). HSP90 served as a loading control. Quantification relative to loading control (*right*). (*C*) Immunoblot for SOD1 and SOD2 during AP (*left*). HSP90 served as a loading control. Quantification relative to loading control (*right*). (*D*) Immunoblot for CAT and PRDX1 during AP (*left*). HSP90 served as a loading control. Quantification relative to loading control (*right*). (*E*) Immunoblot for PRDX2 and PRDX6 during AP (*left*). HSP90 served as a loading control. Quantification relative to loading control (*right*). (*F*) Immunoblot for GPX1 and GPX2 during AP (*left*). HSP90 served as a loading control. Quantification relative to loading control (*right*). (*G*) Immunoblot for GPX8 and GSTM1 during AP (*left*). HSP90 served as a loading control. Quantification relative to loading control (*right*). (*H*) Immunoblot for GSTT2 during AP (*left*). HSP90 served as a loading control. Quantification relative to loading control (*right*). TXNRD1 (*B*) and PRDX1 (*D*) blots, as well as CAT (*D*) and PRDX6 (*E*) blots, were derived from the same membranes and lane orders; the HSP90 loading controls are reused for both panel pairs. All results are means ± SD. *P* values were calculated using 1-way ANOVA followed by Dunnett’s post hoc correction for multiple comparisons. ns, not significant; ∗∗, significant at *P* < .01.

To identify changes in antioxidant defense during AP, we induced pancreatitis in wild-type mice and collected RNA at 0 hours, 8 hours, and 24 hours. We then performed RNA sequencing (RNA-seq) analysis and specifically focused on genes implicated in ROS defense. In our dataset, 22 genes (of 78 genes detected in the RNA-seq and associated with Gene Ontology [GO] term 16209 “antioxidant activity”) were significantly differentially expressed in at least 1 of the 2 timepoints after multiple testing correction compared with the basal timepoint (Figure 1*A*; Supplementary Table 1).

The most significantly upregulated gene was thioredoxinreductase 1 at 8 hours vs basal (*Txnrd1*, upregulation 8-fold). Other significantly upregulated genes were *Srxn1*, *Gstp3*, and *Prdx1* (10-fold, 8-fold, and 1.4-fold, respectively). On the other hand, some components of the GSH and peroxiredoxin system were significantly downregulated (for example, *Prdx3* and *Gstk1* [2-fold and 2.1-fold, respectively]).

To identify potential upstream regulators of the observed transcriptional changes, we performed motif enrichment analysis using RcisTarget on the 15 highest-ranked genes annotated with GO:0016209 at 8 hours (Supplementary Table 2). Although the top-ranked motif based on area under the curve (AUC) and normalized enrichment score (NES) corresponded to a heterodimeric binding site for C/EBPβ and AP-1, this likely reflects a general stress response. In contrast, 5 of the top 10 enriched motifs were associated with nuclear factor erythroid 2-related factor 2 (NRF2), suggesting activation of NRF2-dependent regulatory pathways more specifically related to antioxidant gene regulation.

To assess changes of key antioxidant proteins during AP, we performed a more detailed analysis by immunoblots (Figure 1*B–H*) with select members of the thioredoxin, the GSH, the peroxiredoxin, and the superoxide dismutase systems. Here, as well, we detected a significant increase in TXNRD1 expression, whereas TXNRD2 remained mostly unchanged over time. Cytosolic SOD1 decreased at 3 and 8 hours, whereas mitochondrial SOD2 did not change significantly. Catalase increased significantly at 8 and 24 hours, and the peroxiredoxins 1, 2 and 6 did not change significantly. Components of the GSH system showed changes mainly with a significant decrease in GPX1 at all time points after induction and a temporary significant decrease of GPX2.

In summary, we detected significant changes both in RNA and protein expression of antioxidant proteins during experimentally induced AP. We then decided to focus more specifically on Txnrd1 and the GSH system.

### Txnrd1 Knockout Mice Show Normal Pancreatic Development and Function Under Baseline Conditions

**Figure 2:**
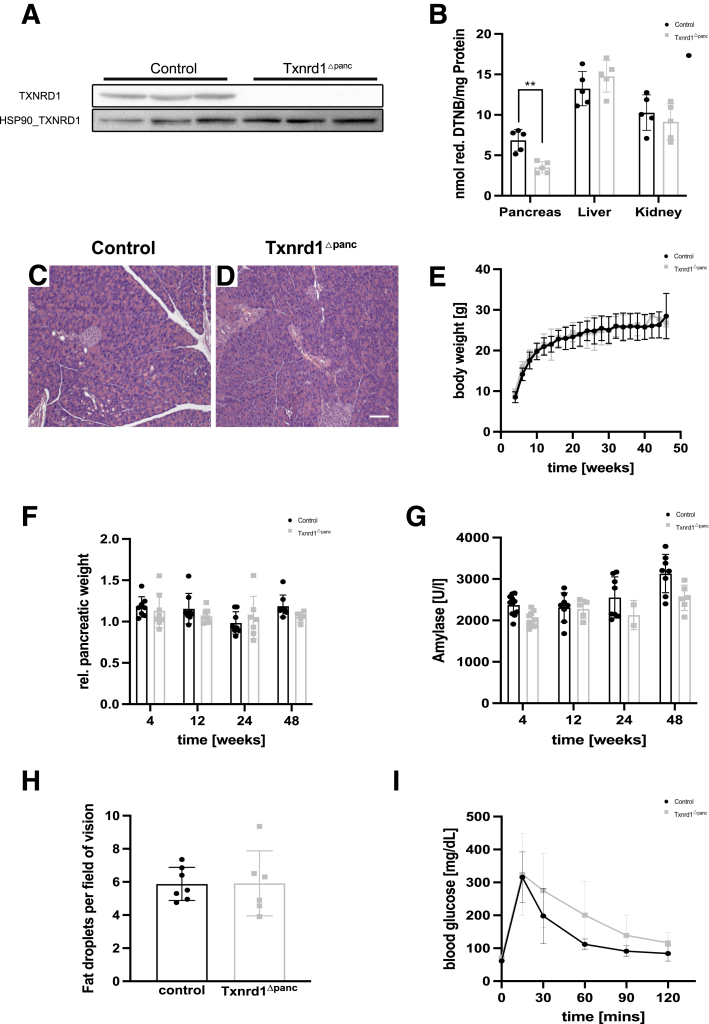
**Characterization of *Txnrd1*^Δpanc^ animals.** (*A*) Immunoblot for TXNRD1 in 10-week-old control and *Txnrd1*^Δpanc^ mice. (*B*) Cytosolic thioredoxin reductase activity of 3-week-old control and Txnrd1^Δpanc^ animals in indicated organs. (*C*) Histology (H&E staining) of pancreata of 10-week-old control and (*D*) *Txnrd1*^Δpanc^ mice. (*E*) Body weight of control and *Txnrd1*^Δpanc^ animals at indicated timepoints. (*F*) Relative pancreas weight (to total body weight) of control and *Txnrd1*^Δpanc^ animals at indicated timepoints. (*G*) Serum amylase values of control and *Txnrd1*^Δpanc^ animals at indicated timepoints. (*H***)** Fat droplets in stool of control and *Txnrd1*^Δpanc^ animals. (*I*) Blood glucose values of 1-year-old control and Txnrd1^Δpanc^ animals after glucose tolerance test. All results are means ± SD. *P* value using unpaired *t*-test. ∗∗, significant at *P* < .01.

We generated *Txnrd1*^Δpanc^ mice and observed them for overt phenotypic changes. The protein level of Txnrd1 was significantly decreased in *Txnrd1*^Δpanc^ mice (Figure 2*A*), and thioredoxin reductase activity was significantly decreased in the pancreas of these mice (Figure 2*B*), whereas the enzymatic activity in liver and kidney was not significantly changed. Pancreatic histology at 10 weeks appeared similar between knockout and control mice (Figure 2*C, D*). In addition, growth and relative pancreatic weight were not significantly different from controls at multiple timepoints over 1 year (Figure 2*E and F*). In addition, serum amylase levels were not significantly different to those from control animals over time (Figure 2*G*). To assess for exocrine insufficiency, stool samples of animals were analyzed after feeding with a high-fat diet. Here, we did not find an increase in lipid droplets (Figure 2*H*). Endocrine function was then investigated using a glucose-tolerance test. Basal blood glucose values and the increase after glucose administration were similar in both groups. In both groups, glucose levels then decreased; however, this decline was observed to be slower in *Txnrd1*^Δpanc^ mice, although this difference was not significant (Figure 2*I*).

**Figure 3:**
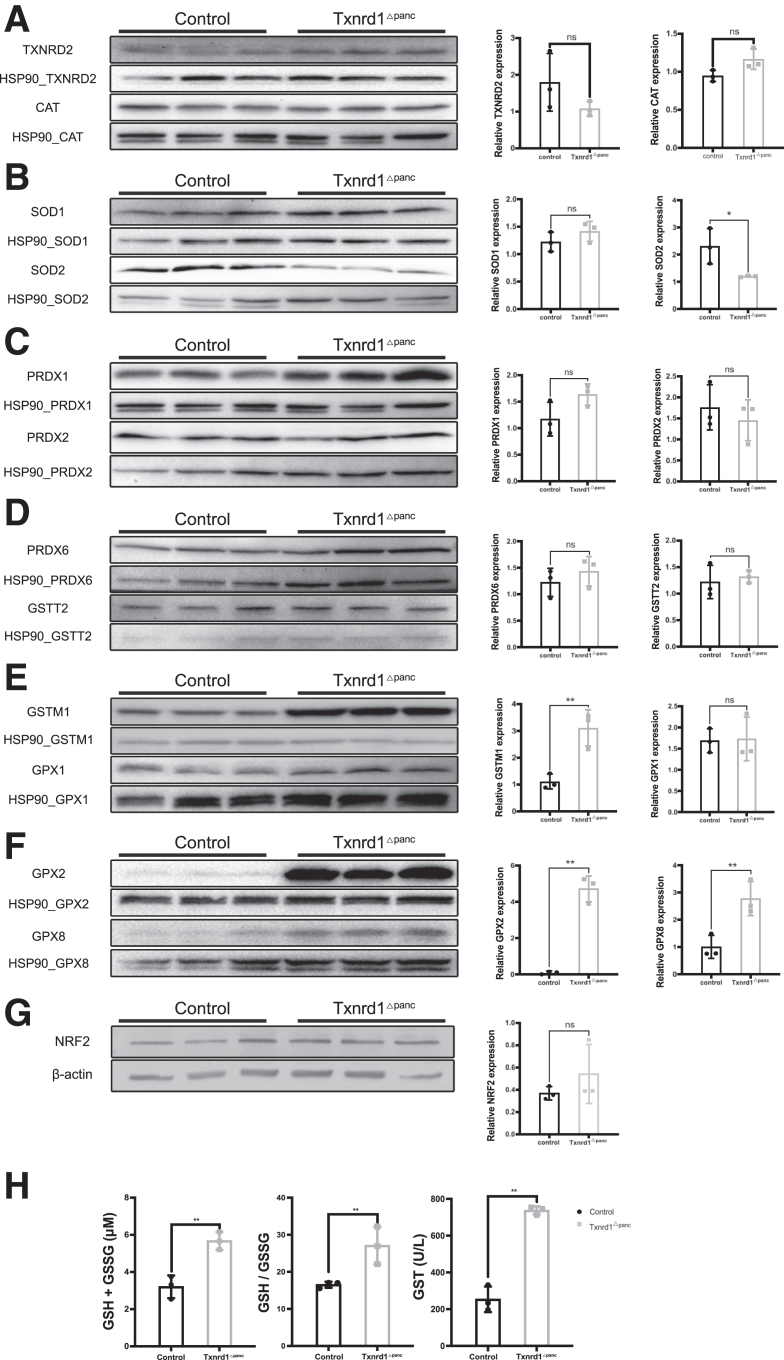
**The glutathione system compensates for loss of Txnrd1.** Immunoblots of antioxidant proteins in the pancreas of 10-week-old control and *Txnrd1*^Δpanc^ mice. (*A*) Immunoblot for TXNRD2 and CAT (*left*). HSP90 served as a loading control. Quantification relative to loading control (*right*). (*B*) Immunoblot for SOD1 and SOD2 (*left*). HSP90 served as a loading control. Quantification relative to loading control (*right*). (*C*) Immunoblot for PRDX1 and PRDX2 (*left*). HSP90 served as a loading control. Quantification relative to loading control (*right*). (*D*) Immunoblot for PRDX6 and GSTT2 (*left*). HSP90 served as a loading control. Quantification relative to loading control (*right*). (*E***)** Immunoblot for GSTM1 and GPX1 (*left*). HSP90 served as a loading control. Quantification relative to loading control (*right*). (*F*) Immunoblot for GPX2 and GPX8 (*left*). HSP90 served as a loading control. Quantification relative to loading control (*right*). (*G*) Immunoblot for NRF2. β-Actin served as a loading control. Quantification relative to loading control (*right*). (*H*) Total GSH concentration, GSH/GSSG ratio, and GST activity in pancreatic tissue. All results are means ± SD. *P* values using unpaired *t*-test. ns, not significant; ∗, significant at *P* < .05; ∗∗, significant at *P* < .01.

The expression of TXNRD2, catalase, and peroxiredoxins 1, 2, and 6 was not significantly different between knockouts and controls (Figure 3*A–D*). However, several components of the glutathione system were significantly increased in *Txnrd1*^Δpanc^ mice, namely GSTM1, GPX2, and GPX8 (Figure 3*E, F*). Nrf2 has previously been shown to be upregulated secondary to *Txnrd1* deletion^4^; however, we could not detect such a change in our samples (Figure 3*G*).

We then asked whether the observed changes in components of the GSH system in *Txnrd1*^Δpanc^ mice resulted in measurable changes of total GSH and ratio of reduced to oxidized GSH. Notably, both GSH + GSSG and the ratio of GSH to GSSG were significantly increased in *Txnrd1*^Δpanc^ mice (Figure 3*H*). To assess global GSH conjugation activity, a GST-CDNB assay was performed, revealing a significant elevation in conjugation capacity in *Txnrd1*^Δpanc^ pancreatic tissue (Figure 3*H*).

### Txnrd1 Deletion Causes a Marginally More Severe AP

**Figure 4:**
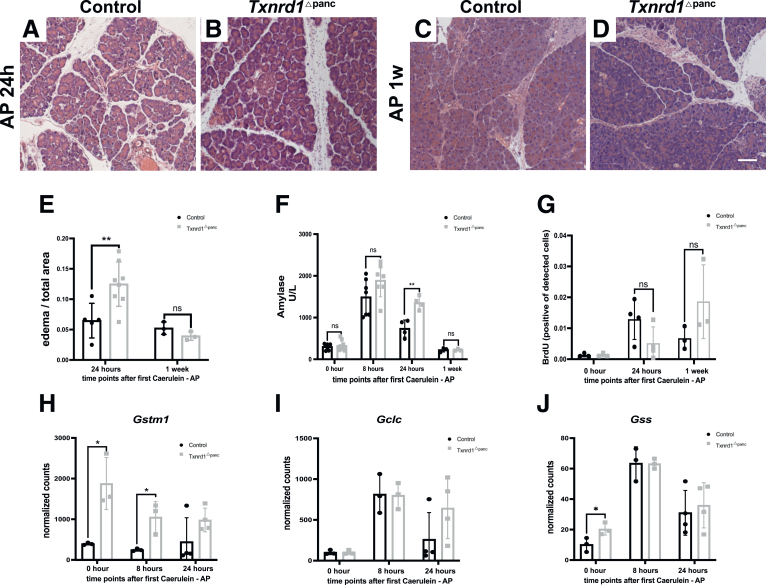
**AP in *Txnrd1*^Δpanc^ animals.** (*A–D*) Histology (H&E staining) of pancreata after cerulein administration. Scale bar = 100 μm. (*E*) Ratio of edema to total pancreas area after cerulein administration. (*F*) Serum amylase levels after cerulein administration at indicated time points. (*G*) BrdU quantification after cerulein administration; each dot represents one mouse. RNA expression of *Gstm1* (*H*), *Gclc* (*I*), and *Gss* (*J*) at basal condition and after cerulein administration. All results are means ± SD. *P* values using unpaired *t*-test. ns, not significant; ∗, significant at *P* < .05; ∗∗, significant at P < .01.

Given the previously observed changes of Txnrd1 during AP, we challenged *Txnrd1*^Δpanc^ mice with experimentally induced AP. The amount of edema formation at 24 hours was significantly higher in *Txnrd1*^Δpanc^ mice, but an increase of necrosis or infiltration was not seen (Figure 4*A–B and E*). In both groups, pancreatic histology after 1 week of recovery was unremarkable (Figure 4*C–D*). Serum levels of amylase were significantly higher in *Txnrd1*^Δpanc^ mice at 24 hours after induction but were similar between knockouts and controls at baseline and after 1 week (Figure 4*F*).

Bromodeoxyuridine (BrdU) quantification (Figure 4*G*) revealed increased proliferation in both control and *Txnrd1*^Δpanc^ mice following AP induction, although the difference between groups was not statistically significant. Interestingly, *Gstm1* expression was significantly higher in knockout mice at baseline and at 8 hours after pancreatitis induction (Figure 4*H*). The expression of *Gclc* (Fig. 4I) increased in both control and *Txnrd1* knockout mice at 8 hours post-AP induction, reaching comparable levels. At 24 hours, *Gclc* expression remained elevated but showed a nonsignificant trend toward higher levels in control mice. In contrast, *Gss* expression (Figure 4*J*) was significantly elevated in *Txnrd1* knockout mice at baseline. Following AP induction, *Gss* expression increased similarly in both groups at 8 hours. By 24 hours, expression levels remained above baseline but were reduced compared with the 8-hour peak, with no significant differences observed between genotypes.

**Figure 5:**
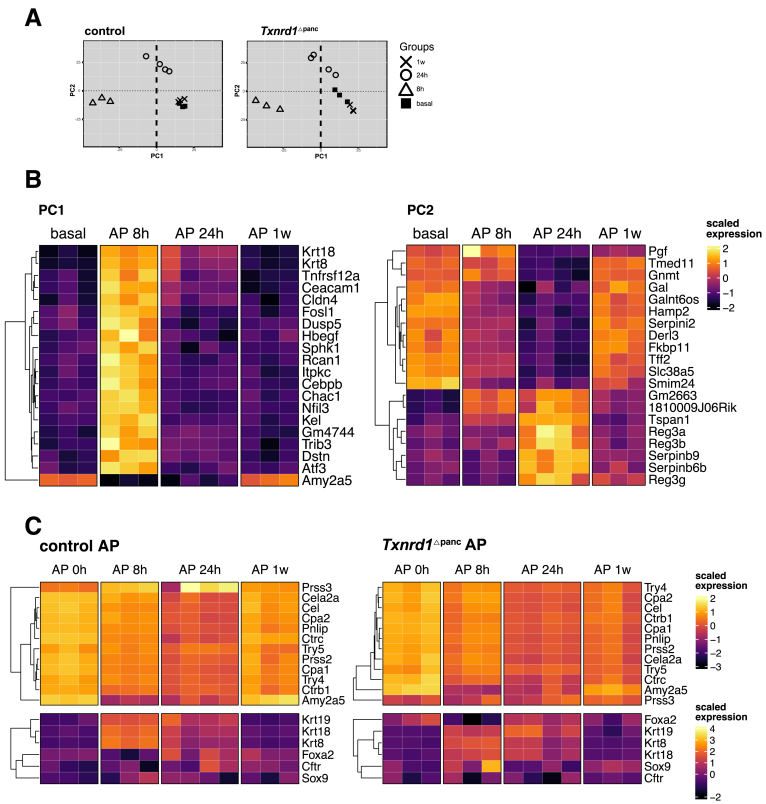
**PCA during pancreatitis in control and *Txnrd1*^Δpanc^ mice.** (*A*) PCA plots for the RNA-seq analysis in control and *Txnrd1*^Δpanc^ animals. The explained amount of the total variance of the full data set is shown for each principal component (PC1–2). (*B*) Top 20 genes contributing to PC1 and PC2 based on PCA loadings from transcriptomic data of control mice during AP. (*C*) Expression of acinar and ductal specific genes at indicated time points in control and *Txnrd1*^Δpanc^ mice. Expression values were scaled across the full RNA-seq dataset to ensure comparability with other heatmaps.

We then performed RNA-seq and principal component analysis (PCA) in these groups and found a clear separation of samples from baseline, at 8 hours, and at 24 hours in both groups (Figure 5*A*). Samples after 1 week did not clearly separate from the baseline samples in both knockouts and controls, which agrees with the resolution of changes seen on the histology after 1 week.

We further examined the principal components of gene expression in control mice during AP by analyzing the loadings of PC1 and PC2 (Figure 5*B*). PC1 predominantly reflected temporal changes associated with acinar-to-ductal metaplasia. At 8 hours, we observed a pronounced upregulation of ductal markers including *Krt19*, *Krt8*, and *Cldn4*. This increase was transient, declining substantially by 24 hours and remaining low at the 1-week time point. Concurrently, *Amy2a5* expression was markedly downregulated at 8 hours and showed recovery only at 1 week. In contrast, PC2 captured a distinct pattern characterized by a transient upregulation of *Reg3g*, *Reg3b*, and *Reg3a* at 24 hours, suggestive of an intermediate regenerative response phase.

Given these transcriptional dynamics, we next examined the expression profiles of acinar- and ductal-specific marker genes in both control and *Txnrd1*^Δpanc^ mice during AP (Figure 5*C*). Acinar gene expression was uniformly suppressed at 24 hours postinduction in both genotypes, consistent with transient acinar dedifferentiation. By 1 week, acinar marker expression was notably higher in control mice, indicating a more complete recovery of acinar identity compared with *Txnrd1*^Δpanc^ mice. In contrast, the expression levels of ductal markers remained comparable between the 2 groups across all time points.

Taken together, we did not see a profound effect of *Txnrd1* deletion on pancreatic phenotype and only a mild effect on the course of AP. The increased expression of components of the GSH system and the increases in GSH content in *Txnrd1*^Δpanc^ mice led us to the hypothesis that the GSH system might compensate for the loss of *Txnrd1*.

### Glutathione Compensates for Loss of Txnrd1

**Figure 6:**
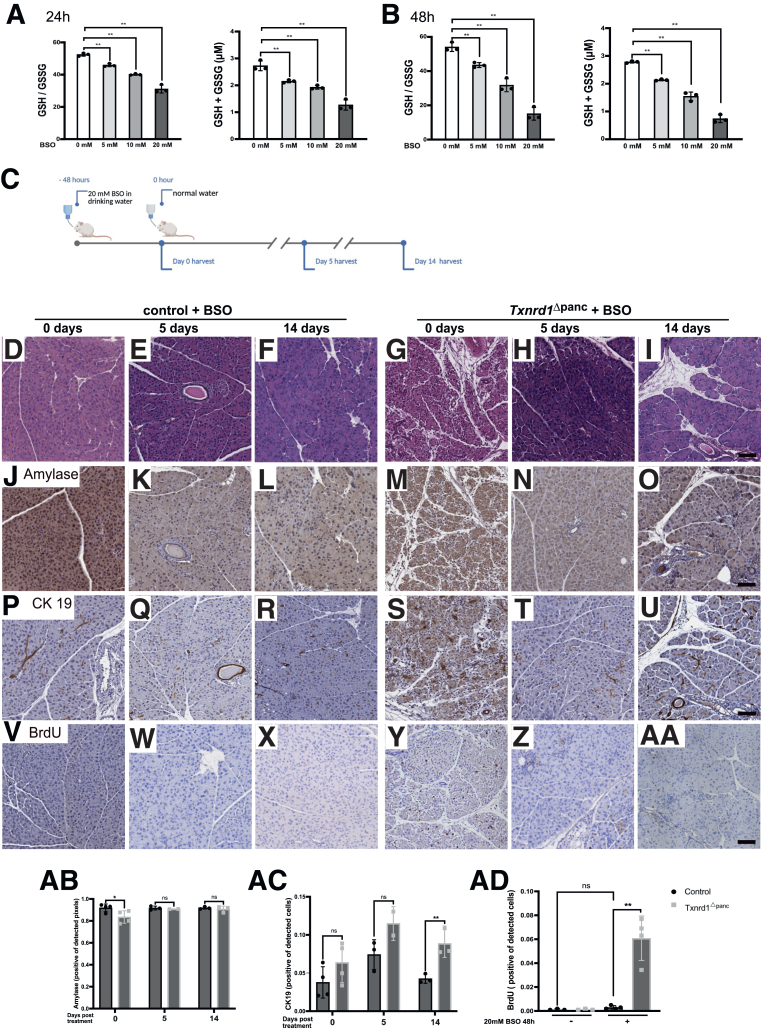
**Expression of antioxidant components after treatment with BSO.** (*A*) GSH/GSSG ratio and total GSH concentration in pancreata in wild-type animals after 24 hours of 0, 5, 10, and 20 mM BSO treatment. (*B*) GSH/GSSG ratio and total GSH concentration in pancreata in wild-type animals after 48 hours of 0, 5, 10, and 20 mM BSO treatment. (*C*) Schedule of glutathione depletion. H&E stainings of pancreata after (*D, G*) BSO treatment, (*E, H*) 5 days after BSO treatment, and (*F, I*) 14 days after BSO treatment. Amylase IHC stainings of pancreata after (*J, M*) BSO treatment, (*K, N*) 5 days after BSO treatment, and (*L, O*) 14 days after BSO treatment. CK19 IHC stainings of pancreata after (*P, S*) BSO treatment, (*Q, T*) 5 days after BSO treatment, and (*R, U*) 14 days after BSO treatment. BrdU IHC stainings of pancreata after (*V, Y*) BSO treatment, (*W, Z*) 5 days after BSO treatment, and (*X, AA*) 14 days after BSO treatment. Scale bar = 100 μm. (*AB*) Quantification of amylase staining intensity; each dot represents 1 mouse. (*AC*) Quantification of CK19-positive cells; each dot represents 1 mouse. (*AD*) Quantification of proliferation at baseline and BSO treatment; each dot represents 1 mouse. All results are means ± SD. *P* values in *A and B* were calculated using 1-way ANOVA followed by Dunnett’s post hoc correction for multiple comparisons; *P* values in *AB–AD* were calculated using unpaired *t*-test. ns, not significant; ∗, significant at *P* < .05; ∗∗, significant at *P* < .01.

To examine this idea, we used the GSH synthesis inhibitor buthionine sulfoximine (BSO) to deplete the glutathione pool. We found a dose of 20 mM in the drinking water, administered over 48 hours, to best deplete both total GSH and the ratio of reduced to oxidized GSH (Figure 6*A–B*) without any changes in animal well-being.

Both control and *Txnrd1*^Δpanc^ mice then received BSO according to these predetermined conditions (Figure 6*C*).

After BSO treatment in control animals, histologic examination revealed no notable changes; specifically, areas of necrosis, edema, or inflammatory infiltrates were not observed (Figure 6*D*). At both 5 days and 14 days after BSO treatment, pancreatic histology of control animals was unremarkable (Figure 6*E–F*).

In *Txnrd1*^Δpanc^ mice, however, BSO treatment resulted in large areas of necrosis and focal infiltration by inflammatory cells (Figure 6*G*). However, fatty necrosis of adjacent tissue was not seen. Five days after BSO treatment, the pancreas of *Txnrd1*^Δpanc^ mice had largely recovered, and necrosis was reduced greatly, with some areas of infiltrating cells persisting (Figure 6*H*). Two weeks after BSO treatment, acinar tissue had greatly recovered in *Txnrd1*^Δpanc^ mice, but areas of infiltrating cells and unrecovered acinar cells remained. (Figure 6*I*).

To further characterize the response to BSO treatment, we performed immunohistochemical (IHC) stainings for amylase, CK19, and BrdU at the same timepoints. Amylase staining (Figure 6*J–O*; quantification Figure 6*A–B*) revealed a significant reduction in *Txnrd1*^Δpanc^ mice after 48 hours of BSO treatment, but no appreciable differences were observed between genotypes at later stages of recovery. In contrast, CK19 staining (Figure 6*P–U*; quantification Figure 6*A, C*) demonstrated a marked increase in ductal marker-positive cells in *Txnrd1*^Δpanc^ mice at 2 weeks after 48 hours of BSO, suggesting enhanced ductal reprogramming.

BSO treatment dramatically increased proliferation (as determined by BrdU staining, Figure 6*V–AA*) in the pancreas in both control and *Txnrd1*^Δpanc^ mice (2-fold and 45-fold, respectively), and proliferation in BSO-treated *Txnrd1*^Δpanc^ mice was significantly higher (fold-change 20×) compared with BSO-treated controls (Figure 6*A, D*).

**Figure 7:**
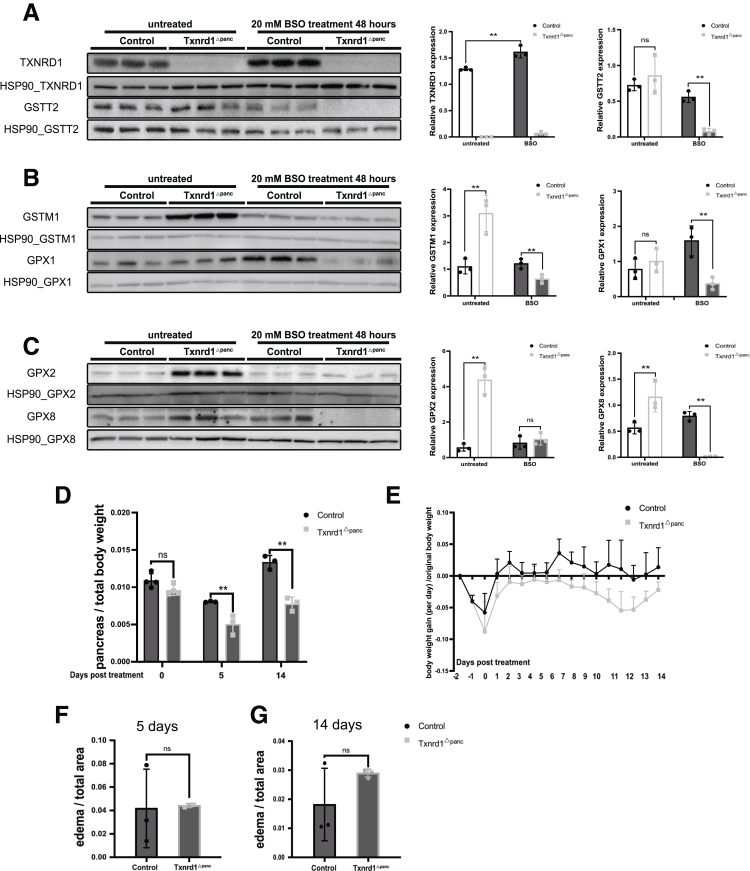
**BSO affects the glutathione system more severely in Txnrd1^Δpanc^ mice.** (*A*) Immunoblots for TXNRD1 and GSTT2 after BSO treatment (*left*). HSP90 served as a loading control. Quantification relative to loading control (*right*). (*B*) Immunoblots for GSTM1 and GPX1 after BSO treatment (*left*). HSP90 served as a loading control. Quantification relative to loading control (*right*). (*C*) Immunoblots for GPX2 and GPX8 after BSO treatment (*left*). HSP90 served as a loading control. Quantification relative to loading control (*right*). (*D*) Relative pancreatic weight after glutathione depletion. (*E*) Change in body weight after glutathione depletion. Units = days post treatment. Ratio of edema to total pancreas area after 5 days (*F*) and 14 days (*G*) of BSO treatment. All results are means ± SD. *P* values were calculated using unpaired *t*-test. ns, not significant; ∗∗, significant at *P* < .01.

In addition, we performed immunoblot analysis from *Txnrd1*^Δpanc^ and control mice 48 hours after BSO administration (Figure 7*A–C*). In *Txnrd1*^Δpanc^ mice, basal upregulation of the antioxidant proteins GSTM1, GPX2, and GPX8 was abrogated following BSO treatment, whereas in control mice, expression of these proteins remained largely unchanged. In control mice, BSO treatment induced TXNRD1 expression and expression of GPX1 and GPX8.

In *Txnrd1*^Δpanc^ mice, relative pancreatic weight was significantly lower than that of control mice both 5 days and 14 days after BSO therapy (Figure 7*D*). In addition, animals from both groups lost 5% to 10% of body weight during BSO treatment. This weight loss recovered 1 to 2 days after treatment, with control animals not losing weight after that. *Txnrd1*^Δpanc^ mice, however, started losing weight again 1 week after BSO treatment and then started to recover around day 12 (Figure 7*E*).

Pancreatic edema quantification in BSO-treated control and *Txnrd1*^Δpanc^ mice was not significantly different between groups at 5 (Figure 7*F*) or 14 days (Figure 7*G*) after BSO administration.

### Glutathione Depletion and Txnrd1 Deletion Result in Loss of Pancreatic Tissue During AP

**Figure 8:**
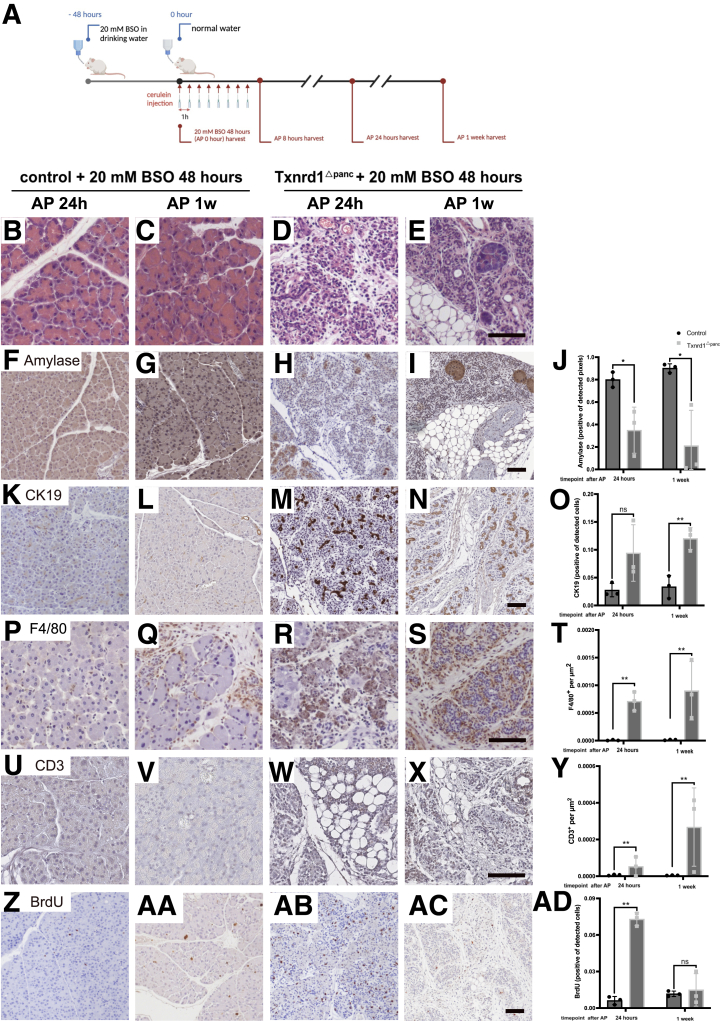
**The role of glutathione in the *Txnrd1*-deficient pancreas during AP.** (*A*) Schedule of glutathione depletion with AP. H&E stainings of pancreata on (*B, D*) BSO treatment combined with AP 24 hours after induction, (*C, E*) BSO treatment combined with AP at 1 week. Amylase IHC stainings of pancreata after (*F, H*) BSO treatment combined with AP at 24 hours, (*G, I*) BSO treatment combined with AP at 1 week, quantification in (*J*). CK 19 IHC stainings of pancreata after (*K, M*) BSO treatment combined with AP at 24 hours, (*L, N*) BSO treatment combined with AP at 1 week, quantification in (*O*). F4/80 IHC stainings of pancreata after (*P, R*) BSO treatment combined with AP at 24 hours, (*Q, S*) BSO treatment combined with AP at 1 week, quantification in (*T*). CD3 IHC stainings of pancreata after (*U, W*) BSO treatment combined with AP at 24 hours, (*V, X*) BSO treatment combined with AP at 1 week, quantification in (*Y*). BrdU IHC stainings of pancreata after (*Z, AB*) BSO treatment combined with AP at 24 hours, (*AA, AC*) BSO treatment combined with AP at 1 week, quantification in (*AD*). Scale bar = 100 μm. Each dot represents 1 mouse. All results are means ± SD. *P* values were calculated using unpaired *t*-test. ns, not significant; ∗, significant at *P* < .05; ∗∗, significant at *P* < .01.

We then set out to investigate the impact of combined depletion of GSH and deletion of *Txnrd1* on the course of AP. The experimental design is depicted in Figure 8*A*.

In control animals, we detected increased edema and macrophage infiltration at 24 hours and a mild increase in macrophage infiltration and proliferation after 1 week.

In *Txnrd1*^Δpanc^ animals, there was a profound loss of acinar tissue at 24 hours (Figure 8*H*). This depletion of acinar cells did not recover after 1 week as evidenced by amylase staining (Figure 8*I*); only ductal structures remained. In addition, a substantial infiltration by macrophages was evident (Figure 8*R–S*).

Twenty-four hours after AP induction, proliferation in the pancreas of BSO-treated *Txnrd1*^Δpanc^ mice increased to 7-fold compared with BSO-treated controls after AP induction but was almost absent at 1 week (Figure 8*Z–AC*; for quantification Figure 8*A, D*).

**Figure 9:**
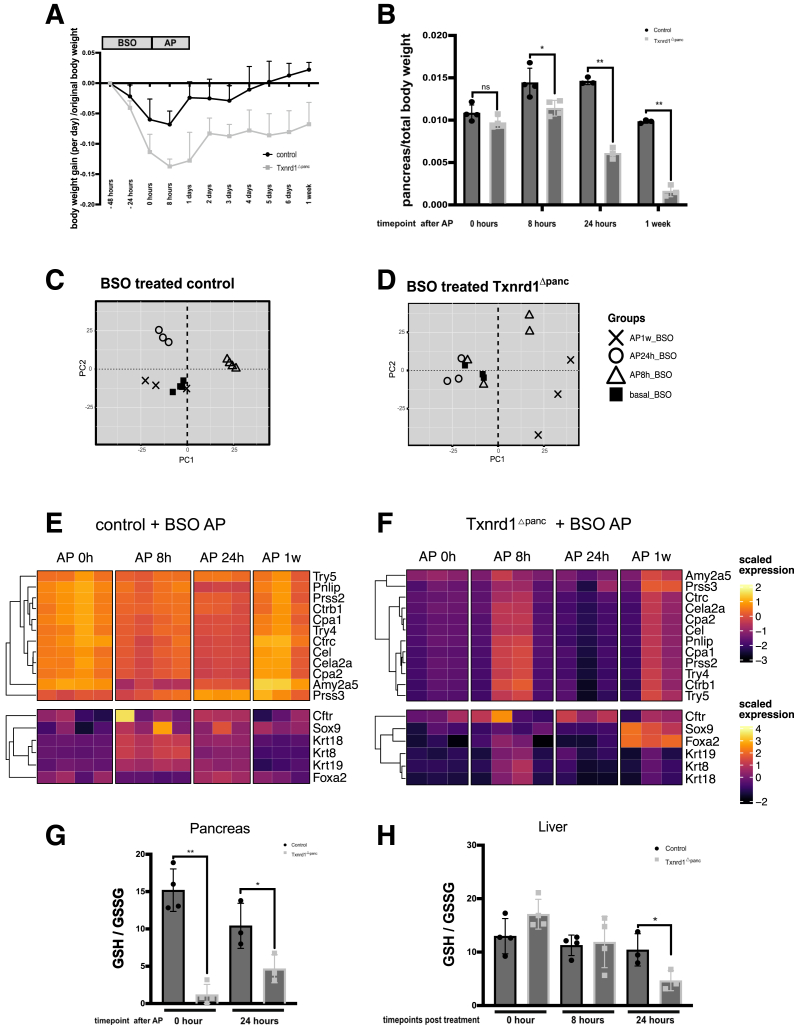
**Combined glutathione depletion and *Txnrd1* deletion severely affects pancreatic homeostasis.** (*A*) Body weight changes during glutathione depletion with AP at indicated timepoints. (*B*) Relative pancreas weight (to total body weight) of control and *Txnrd1*^Δpanc^ animals after BSO treatment combined with cerulein administration. PCA scores plot for the RNA-seq analysis after AP in (*C*) BSO-treated control and (*D*) BSO-treated *Txnrd1*^Δpanc^ animals. The explained amount of the total variance of the full data set is shown for each principal component (PC1–2). Expression of acinar and ductal specific genes at indicated time points in (*E*) control and (*F*) Txnrd1^Δpanc^ mice. Expression values were scaled across the full RNA-seq dataset to ensure comparability with other heatmaps. (*G*) GSH/GSSG ratio in pancreata in control animals and *Txnrd1*^Δpanc^ animals after 48 hours of 20 mM BSO treatment and combined with AP 24 hours. (*H*) GSH/GSSG ratio in liver tissues after 48 hours of 20 mM BSO treatment and after AP induction. All results are means ± SD. *P* values were calculated using unpaired *t*-test: ns, not significant; ∗, significant at *P* < .05; ∗∗, significant at *P* < .01.

BSO treatment and AP resulted in weight loss in both control and knockout mice, but to a greater extent in *Txnrd1*^Δpanc^ mice. Control mice began to recover more quickly, with positive weight gain by day 6. In contrast, although *Txnrd1*^Δpanc^ mice also demonstrated recovery over time, they lost weight throughout the observation period of 1 week, indicating an incomplete recovery compared with controls (Figure 9*A*).

Relative pancreatic weight was significantly lower in BSO-treated *Txnrd1*^Δpanc^ mice already at 8 hours after AP induction. This difference to control animals increased significantly over time, to a relative pancreatic weight of 41% of that of controls at 24 hours, and at 1 week after AP, the relative pancreatic weight of *Txnrd1*^Δpanc^ mice was only 13% of that of controls (Figure 9*B*).

RNA-seq and PCA in these mice showed similar changes over time in BSO-treated control animals (Figure 9*C*) as shown for AP in control animals without BSO (Figure 5*A*): a clear separation of samples from baseline, at 8 hours, and at 24 hours, and an overlap of samples from 1 week with baseline samples, which agrees with the unremarkable pancreatic histology in BSO-treated control mice after 1 week.

In BSO-treated *Txnrd1*^Δpanc^ animals, however, PCA analysis demonstrated that samples from 8 and 24 hours did not separate clearly from baseline samples (Figure 9*D*). In addition, the samples from 1 week after induction of AP were distant from the baseline samples, which is in line with the severely impaired pancreas with loss of acinar cells and increased macrophage infiltration.

The expression profiles of acinar- and ductal-specific marker genes in BSO-treated control and *Txnrd1*^Δpanc^ mice during acute pancreatitis differed markedly (Figure 9*E, F*). BSO-treated control mice retained an expression pattern closely resembling that of untreated control and *Txnrd1*^Δpanc^ mice during AP. In contrast, acinar marker expression in BSO-treated *Txnrd1*^Δpanc^ mice was profoundly suppressed at all time points, with only minor recovery observed in a subset of animals at 8 hours and 1 week. Notably, the ductal markers *Sox9* and *Foxa2* were upregulated at 1 week exclusively in the knockout group—a response absent in all other conditions—suggesting a shift toward a ductal phenotype under impaired redox regulation.

In the pancreas, the GSH/GSSG ratio after BSO treatment was significantly lower in *Txnrd1*^Δpanc^ animals than in control animals (13-fold reduction). Interestingly, that ratio increased slightly at 24 hours, but decreased in control animals, with the difference between control and *Txnrd1*^Δpanc^ animals remaining significant (at 2.2-fold) (Figure 9*G*)

We also measured the GSH/GSSG ratio in livers after BSO treatment and after AP induction (Figure 9*H*). The liver GSH/GSSG ratio was not significantly different between controls and *Txnrd1*^Δpanc^ animals just after BSO treatment and early after BSO treatment and AP induction (8 hours). However, at 24 hours after AP induction, the ratio was 2-fold reduced in *Txnrd1*^Δpanc^ mice compared with controls, indicating systemically increased oxidative stress in BSO-treated *Txnrd1*^Δpanc^ animals.

These results show that the GSH system compensates for the loss of thioredoxin reductase 1 in vivo and that additional inhibition of GSH abolishes the required antioxidant response during AP.

## Discussion

Our findings shed light on changes in antioxidant responses during experimentally induced AP. Notably, over 28% of all genes with antioxidant functions exhibited significant changes within the first 24 hours of AP. Motif analysis of the upregulated genes suggested potential involvement of NRF2, a key regulator of oxidative stress responses. Among these, the upregulation of *Txnrd1* and profound alterations in components of GSH metabolism prompted us to investigate both systems in greater detail.

Interestingly, pancreas-specific deletion of *Txnrd1* did not lead to significant alterations in pancreatic histology and function under baseline conditions. Similar findings have been made in other organs. For example, deletion of *Txnrd1* in cardiomyocytes did not affect heart development or function.^5^ However, *Txnrd1* deletion has been shown to play a critical role in the expansion of CD4−CD8−thymocytes^6^ and for B-cells.^7^ In hepatocytes, a compensatory upregulation of Nrf2 along with several components of the GSH pathway was observed.^4^ Although we did not detect a NRF2 upregulation, we did find an increase in GSH levels and higher GSH/GSSG ratio, higher GST activity, and *Gstm1* upregulation, suggesting a compensatory mechanism.

Although *Gss* expression was elevated at basal condition, both *Gss* and *Gclc* rose similarly in both groups during AP. The sustained *Gstm1* upregulation in *Txnrd1*^Δpanc^ mice suggests that enhanced GSH metabolism in this model is primarily driven by increased conjugation activity rather than de novo synthesis.

Following AP induction, *Txnrd1*^Δpanc^ mice showed significantly more edema and higher serum amylase levels compared with controls. However, pancreatic histology, gene expression, and serum amylase levels returned to normal within 1 week.

Depleting GSH in control animals did not significantly alter pancreatic histology or the course of AP, suggesting that the glutathione depletion alone may not be responsible for the increased severity of AP.^8^^,^^9^ Notably, 2 studies examining the intravenous administration of N-acetylcysteine (NAC) during AP found no therapeutic benefit.^10^^,^^11^ In contrast, 2 clinical trials^12^^,^^13^ reported a significant reduction in the incidence of post-endoscopic retrograde cholangiopancreatography (ERCP) pancreatitis following oral NAC administration. Although both trials were conducted by the same research group, the consistent findings across distinct cohorts suggest a context-dependent protective effect of NAC, possibly related to increased local effects with oral administration. These observations highlight that although systemic NAC has shown limited efficacy in treating ongoing AP, antioxidant interventions might offer prophylactic benefit if applied prior to the onset of inflammatory damage. However, further studies are warranted to delineate the therapeutic window, route of administration, and mechanistic targets of redox-based interventions in AP.

In contrast, in *Txnrd1*^Δpanc^ animals, glutathione depletion caused extensive necrosis and significantly increased proliferation. These findings suggest a functional redundancy between the thioredoxin and GSH system. For example, TXNRD1 was upregulated in response to glutathione depletion, whereas GSH levels increased after *Txnrd1* deletion. This reciprocal relationship has also been observed in B-lymphocytes,^7^ and studies in GSH-deficient mice demonstrated an upregulation of TXNRD1.^14^ Additionally, inhibition of thioredoxin reductase with auranofin was reduced by GSH, indicating that GSH can compensate for thioredoxin loss.^15^

Recently, studies of pancreatic *Gpx4* deletion revealed increased pancreatic injury, edema, and mortality following induction of pancreatitis.^16^ These findings suggest that ineffective GPX4 reduction after BSO treatment partly explains the observed phenotype of *Txnrd1*^Δpanc^ mice in our study.

In another approach, RNA-seq analysis of fluorescence-activated cell-sorted acinar cells from mice following cerulein-induced AP identified *Prdx1* and *Txnrd1* as highly upregulated genes.^17^ Interestingly, deletion of *Prdx1* reduced AP severity, suggesting that redox regulation during AP involves complexities beyond simple antioxidant upregulation or depletion.

Combined inhibition of thioredoxin and GSH systems has been proposed as a promising cancer therapy.^18^, ^19^, ^20^, ^21^ However, our results suggest that applying this strategy to patients may result in severe side effects.

In conclusion, the redundancy between the thioredoxin and GSH systems plays a critical protective role in safeguarding the exocrine pancreas against oxidative stress during AP. Further research is essential to better understand the interplay between different antioxidant systems during AP.

## Methods

### Mouse Line Generation and Housing

An established C57BL/6 thioredoxin reductase 1 conditional deletion mouse line was used.^5^ Female mice heterozygous for Ptf1α-Creex1^22^ and homozygous for the floxed allele of Txnrd1 (Txnrd1flox/flox) were bred with male mice homozygous for the floxed allele of Txnrd1. All mice were maintained in a mixed genetic background (C57BL/6/ FVB/129) and housed in a specific pathogen-free facility with a 12 hour:12 hour light:dark cycle and ad libitum access to food and water. Both sexes were included in the analyses. All mice experiments were conducted in accordance with German federal animal protection laws and approved by the institutional animal care and use committee on animal experimentation and the Government of Upper Bavaria.

### Induction of AP in Mice by Cerulein

To induce acute pancreatitis, 10- to 12-week old mice were starved for 9 hours and weighed.^23^ Eight intraperitoneal (ip) injections of a dose of 50 μg/kg body weight of caerulein were given at hourly intervals; t0 was defined as basal condition.

Timepoint analyses for serum, histology, IHC, immunoblot, and gene expression analyses were performed at 0 hours, 8 hours, 24 hours, and 1 week. Amylase was measured using standard procedures.

### Dose and Time Point-finding for Glutathione Depletion in Wild-type Mice

To determine the dose of BSO in drinking water needed for a significant depletion of the GSH pool, wild-type mice were treated with different concentrations (0, 5, 10, and 20 mM) of BSO in the drinking water for 24 and 48 hours. GSH and GSSG were assayed using the GSH/GSSG-Glo assay from Promega (# V6612) according to the manufacturer’s protocol.

### Induction of Glutathione Depletion in Mice by BSO Treatment

To induce glutathione depletion, experimental mice were given BSO in drinking water at 20 mM, the concentration determined in the dose and time point-finding experiment. To monitor the status of the mice, their weight was taken daily; mice were sacrificed at predefined timepoints (0 day, 5 days, and 14 days), and organs were harvested for further analysis.

### Lipid Absorption Test

Mice were kept singles, fasted for 18 hours, and then fed with a 30% high-fat diet (Ssniff EF R/M with 30% fat, Sniff Spezialdiäten GmbH) for 2 consecutive days. Stool samples were collected for 24-hour periods. The stool was homogenized after adding 10 μL distilled water per mg stool. After centrifugation at 200 × g for 5 minutes to remove insoluble material, 5 μL of the supernatant were mixed with 5 μL of freshly prepared and filtered 0.5% Oil Red O solution (5% Oil Red O in 100% propylene glycol), and the whole volume was prepared as stool smears on slides and examined by light microscopy. For quantification, from the center of the coverslip, 20 consecutive fields of sights were counted.

### Intraperitoneal Glucose Tolerance Test

Intraperitoneal glucose tolerance test (IP-GTT) was performed as recommended by.^24^^,^^25^ A CONTOUR XT (Bayer Vital) glucose measurement device together with CONTOUR NEXT sensors (8884487, Bayer Vital) was used for blood glucose measurements.

Briefly, 12 weeks-old mice were morning-fasted for 6 hours. After measuring body weights, initial fasting blood glucose levels were determined with a drop of blood received from the tail veins after small needle puncture. After blood withdrawal, tails were compressed to avoid hematoma.

Next, mice were intraperitoneally injected with 2 g glucose/kg bodyweight using 20% (w/v) glucose solution (2349480, AlleMan Pharma), and blood glucose levels were determined at the times 15 minutes, 60 minutes, 90 minutes, and 120 minutes.

After IP-GTT, mice were returned to standard husbandry conditions and monitored for well-being for at least 2 consecutive days.

### Immunoblots

Proteins were extracted from frozen pancreas tissues in ice-cold RIPA buffer (50 mM Tris pH 7.5, 150 mM NaCl, 1% NP40, 5% sodium deoxycholate, 0.1% SDS, supplemented with proteinase inhibitor complete Mini [Roche]). Protein concentrations were measured using Pierce BCA Protein Assay Kit (23225, ThermoFisher), according to manufacturer’s instructions. Protein lysates were loaded onto 15% SDS-polyacrylamide gel and transferred to a nitrocellulose membrane. Membranes were incubated in blocking buffer for 1 hour, before incubating with their respective primary antibodies (1:1000) and secondary antibodies (horseradish peroxidase [HRP]-conjugated goat anti-rabbit, 1:5000) antibodies. Membranes were then incubated with primer ECL detection reagent to visualize proteins by Molecular Imager Gel DocTM XR system (Bio-Rad). All antibodies used are described in the materials.

### Histology and IHC

The pancreas, liver, duodenum, spleen, and lung were removed and fixed in 4% (w/v) paraformaldehyde at room temperature overnight, and dehydrated with increasing concentrations of ethanol, xylol, and paraffin in an S300 tissue processing unit (Leica). Adjacent 2.5-μm sections were cut and stained with hematoxylin and eosin (H&E) or subjected to IHC. All antibodies used are described in the materials. Signal was detected with the DAB Peroxidase Substrate Kit (SK-4100, Vector) for 2.5 minutes at room temperature according to the manufacturer’s protocol. Tissues were then counterstained by swiftly dipping slides into hematoxylin solution (1.05175.2500, Merck) and subsequent washing under running tap water for 10 minutes. Then, the slides were mounted in pertex embedding medium (41-4012-00, Medite) and sheeted with coverslips (MENZEL-Gläser, BB024032A1, ThermoFisher).

Slides were scanned at the core facility of animal pathology of Zentrum der Präklinischen Forschung, TU München.

The amount of edema was quantified in scanned H&E-stained formalin-fixed, paraffin-embedded (FFPE) slides. Briefly, images were cropped down to the pancreas area using Fiji.^26^ All vessels, ducts, cysts, and fat tissue were also cropped from the image, leaving only acinar tissue and edema. The remaining tissue area was quantified, using the tool “Versatile Wand Tool,” to serve as a reference area. The measured values for the edema area were used as ratios to the total pancreas area.

For CK19, BrdU and F4/80 were quantified with positive cell detection algorithm, and for amylase, with positive pixel count deprecated algorithm from QuPath (version 0.3.2).

### Enzymatic Assays

GSH and GSSG were measured using the GSH/GSSG-Glo assay from Promega (#V6612), according to the manufacturer’s instructions and with the following modification: Pancreas and liver tissue samples were rinsed twice in ice-cold phosphate buffered saline (PBS). Ten volumes of ice-cold 5% SSA (5 g Sulfosalicylic acid in 100 ml deionized water) was added, and the tissue was homogenized until an even suspension was obtained. Samples were centrifuged at 14,000 g at 4°C for 10 minutes. The supernatant was transferred to new tubes, and an equal volume of ice-cold Neutralization Buffer (500 mM HEPES, pH 8) was added. Liver samples were diluted 10-fold in dilution buffer (250 mM HEPES, pH 7.5) to be measured in the linear detection range; pancreas samples were not diluted for measurement. GST enzyme activity was determined using the Glutathione S-transferase Assay Kit from Merck (#MAK453), according to the manufacturer’s instructions.

### RNA-seq

Library preparation for bulk sequencing of poly(A)-RNA was perfomed as described previously.^27^ Barcoded cDNA was synthesized using Maxima RT polymerase (ThermoFisher) with an oligo-dT primer including barcodes, unique molecular identifiers (UMIs), and an adaptor. The cDNA ends were extended using a template switch oligo (TSO), followed by amplification of the full-length cDNA with primers that bind to the TSO site and the adaptor.

NEB UltraII FS kit was used to fragment cDNA. After end repair and A-tailing, a TruSeq adapter was ligated, and 3′-end-fragments were amplified using primers with Illumina P5 and P7 overhangs. Unlike the method described by Parekh et al,^27^ the P5 and P7 sites were exchanged to allow sequencing of the cDNA in read1, whereas barcodes and UMIs were sequenced in read2. The library was sequenced on a NextSeq 500 (Illumina), utilizing 67 cycles for the cDNA for read1 and 16 cycles for read2 to capture barcodes and UMIs. Data processing followed the published Drop-seq pipeline (v1.0) to create sample- and gene-wise UMI tables.^28^ The reference genome for alignment was GRCm38, with transcript and gene definitions according to GENCODE Version M25.

### GO Annotation

Gene annotations corresponding to the GO term "GO:0016209" (antioxidant activity) were obtained using the org.Mm.eg.db annotation package and filtered from the complete set of GO mappings for all genes detected in the dataset.

### Gene Expression Analysis

Differential expression analysis was performed using the DESeq2 package. Adjusted *P* values were calculated using the Benjamini–Hochberg method. For a predefined subset of 78 genes associated with GO:0016209 (‘antioxidant activity’), *P* values from 2 contrasts (8 hours vs basal and 24 hours vs basal) were extracted from the full DESeq2 results. To control the false discovery rate across both timepoints, raw *P* values from both contrasts were combined and adjusted jointly. Genes with a combined adjusted *P* value < .05 were considered significantly regulated in at least one condition. For all other analyses, including PCA and heatmaps, regularized log-transformed (rlog) expression values from DESeq2 were used.

### Motif Enrichment Analysis

Motif enrichment analysis was performed using the RcisTarget package (version 1.23.1).^29^ A combined rank was calculated as log2FC x –log_10_(adjusted *P* value) from the DESeq2 results. From genes annotated with GO:0016209, the 15 most upregulated genes based on this rank were selected. Gene regulatory regions were defined as 500 bp upstream and 100 bp downstream of the transcription start site (TSS). The analysis utilized the precompiled ranking database mm10__refseq-r80__500bp_up_and_100bp_down_tss.mc9nr.genes_vs_motifs.rankings.feather. Motif annotations were derived from motifs-v9-nr.mgi-m0.001-o0.0.tbl. The 10 motifs with the highest NES values were retained.

### Statistical Analysis

Statistical analysis was performed using GraphPad Prism version 9. Unpaired *t*-test was used for comparison between the control group and the knockout group. For all comparisons of conditions/time points to a reference, a 1-way analysis of variance (ANOVA) followed by Dunnett’s post hoc correction for multiple comparisons was performed. Statistical significance was considered when the *P* value was less than .05.
